# Association between homocysteine and conventional predisposing factors on risk of stroke in patients with hypertension

**DOI:** 10.1038/s41598-018-22260-6

**Published:** 2018-03-01

**Authors:** Hui Pang, Bing Han, Qiang Fu, Lin Hao, Zhenkun Zong

**Affiliations:** 1 Department of Cardiology, Xuzhou Central Hospital, Xuzhou Clinical School of Xuzhou Medical University, Affiliated Xuzhou Hospital of Medical College of Southeast University, Xuzhou, Jiangsu China; 2 Department of Urinary Surgery, Xuzhou Central Hospital, Xuzhou, Jiangsu China; 3 Department of Neurosurgery, Affiliated Hospital of Xuzhou Medical University, Xuzhou, Jiangsu China

## Abstract

Previous studies have focused mostly on independent effects of the stroke risk factors, whereas little attention has been paid to interactions between individual factors which may be important for stroke prevention. We collected data related to the patients’ demographic characteristics, history of chronic diseases and lifestyle factors in 2258 patients with primary hypertension. Logistic regression models based on odds ratio (OR) with their associated 95% confidence interval (CI) were used to estimate an independent effect of homocysteine (Hcy) on the risk of stroke but also include the interactions between Hcy and other risk factors. Hcy was associated with an increased OR of the risk of stroke in both hypertension patients (OR, 1.027; 95% CI, 1.016–1.038; P < 0.001) and H-type hypertension patients (OR, 1.026; 95% CI, 1.014–1.037; P < 0.001), after adjustment for potential confounding factors. Among the hypertension participants, three tests of interactions between Hcy and other risk factors were statistically significant: sex, systolic blood pressure and diastolic blood pressure. In conclusion, complexities of the interactions of Hcy stratified by sex and blood pressure need to be considered in predicting overall risk and selecting certain treatments for stroke prevention.

## Introduction

In all 33 province-level administrative units in mainland China, cerebrovascular disease as the most common non-communicable disease, contributed much more to years of life lost in 2013^1^. Stroke is becoming more common among younger people. The burden of stroke is rising in both developed and developing countries due to the resulting neurological impairment. Hypertension is the most important preventable cause of cardiovascular events, with a steep increase with ageing. When compared with other European-American individuals, there is a more close relationship between hypertension and stroke among patients with hypertension in China^2,3^. The China Stroke Primary Prevention Trial (CSPPT) trial was performed in hypertensive patients and demonstrated a positive effect in reducing risk of stroke^4^. However, there is evidence that, the reduction of clinic blood pressure can not lead to an effective reduced incidence of stroke. Hyperhomocysteinemia (HHcy) has been widely accepted as a risk factor for stroke^5^. Furthermore, extensive clinical and preclinical data also support HHcy as an independent predictor of vascular contributions to cognitive impairment and dementia^6^, cerebral vascular resistance^7^, asymptomatic carotid stenosis^8^. Because of the known association of hypertension with both HHcy and stroke, we stratified the analyses by the level of conventional predisposing factors on risk of stroke and determined whether the plasma Hcy levels were associated with stroke. In addition, interactions between elevated Hcy and cardiovascular risk factors were also detected.

## Subjects and Methods

### Patients

This was a retrospective study of 2258 hospitalized patients with primary hypertension recruited from October 2013 to October 2014 from Brain Hospital, Affiliated Hospital of Xuzhou Medical University and department of Cardiology, Xuzhou Central Hospital. Three subtypes of stroke were included: cerebral infarction, cerebral hemorrhage and transient ischemic attack (TIA). Definition of stroke was based on the results of strict neurological examination and computer tomography scans or magnetic resonance imaging according to guidelines for the primary prevention of stroke^9^. Confirmation of hypertension in accordance with the European Society of Hypertension (ESH) Working Group on blood pressure monitoring, is reported in 2014 evidence-based guideline for the management of high blood pressure in adults^10^. Hypertension is defined as values ≥140 mmHg systolic blood pressure (SBP) and/or ≥90 mmHg diastolic blood pressure (DBP), including graded 1(140–159 mmHg SBP and/or 90–99 mmHg DBP), 2(160–179 mmHg SBP and/or 100–109 mmHg DBP), and 3 (≥180 mmHg SBP and/or ≥110 mmHg DBP). In addition, normal blood pressure in patients with hypertension previously controlled on drug therapy were also enrolled in our study. Definition and classification of dyslipidemia is based on 2017 American Association of Clinical Endocrinologists (AACE) and American College of Endocrinology (ACE) guidelines for the management of dyslipidemia and prevention of cardiovascular disease^11^. Triglyceride (TG) levels <150 mg/dL are considered “normal”, with 150 to 199 mg/dL considered “borderline-high” and ≥200 mg/dL considered “high”. Normal values of non-high-density lipoprotein cholesterol (non-HDL-C) are classified as being less than 160 mg/dL, the borderline-high range is 160 to 189 mg/dL, and high risk is ≥190 mg/dL. The World Health Organization (WHO) and numerous other diabetes mellitus (DM) organizations defined the impaired fasting glucose (IFG) cutoff at 6.1 mmol/L. DM is diagnosed based on the fasting plasma glucose (FPG) ≥7.0 mmol/L^12^. The upper limit of normal body mass index (BMI) in adults is 24 kg/m^2^, defined obesity as a BMI ≥28 kg/m^2^, and designate a BMI between these values to be “overweight”^13^. The cutoff values for age are based on recommendations from the Global Burden of Disease Study 2010^14^. Potential participants were excluded if they had a history of acute or chronic infectious diseases, primary glomerulonephritis, arthritis, systemic lupus erythematosus, or malignant tumor. Ethical approval was granted by the Ethics Committee of Xuzhou Central Hospital, and all patients provided written informed consent. All experiments were performed in accordance with relevant guidelines and regulations.

### Data collection procedures

An electronic clinical information system was used to collect data related to the patients’ demographic characteristics, history of chronic diseases and lifestyle factors. The patients underwent a standard physical examination. All fasting venous blood samples and measurement of blood pressure were collected within 24 hours after hospitalization. Blood pressure of patients who had an acute stroke was set to be reassessed when arterial blood pressure and neurological status remained stable. FPG, total cholesterol (TC), TG, high-density lipoprotein cholesterol (HDL-C), and serum creatinine (sCr) levels were measured by using an automatic biochemical analyzer (HITACH 7080, Hitachi Instrument Ltd, Tokyo, Japan). Non-HDL-C is the sum of very low-density lipoprotein cholesterol (VLDL-C) and low-density lipoprotein cholesterol (LDL-C). The estimated glomerular filtration rate (eGFR) was calculated by using the Chronic Kidney Disease Epidemiology Collaboration (CKD-EPI) Study equation^15^. Subjects were considered to be smokers if they had smoked at least 1 cigarette per day for 6 months or 46 months. Drinking was defined as 1 drink at least in the past 30 days. HHcy was defined as a plasme Hcy concentration ≥10 μmol/L. H-type hypertension was defined as subjects with concomitant hypertension and HHcy^16^.

### Statistical Analysis

SPSS17.0 was used to perform the data analysis. Continuous variables were expressed as mean ± standard deviation and categorical variables were presented as frequencies and percentages. Intergroup difference was assessed by *t* test for continuous variables and χ^2^ test for categorical variables. Estimates of relative risks for stroke in hypertension patients with HHcy were based on odds ratio (OR) with their associated 95% confidence interval (CI) from logistic regression. Hcy values were log-transformed to normalize the distribution, with tertile levels as follows: <1.09 (lowest tertile); 1.09–1.23 (middle tertile) and >1.23 (upper tertile). We further examined the detectable interaction between Hcy and stratified factors on predicting the risk of stroke by adding the Hcy × stratified factors cross-product to a logistic regression analysis of the hypertension participants. All *P* values were two-sided and *P* < 0.05 was considered to be statistically significant.

## Results

### Demographic Characteristics

Demographic and risk factor prevalence data for the study sample and the subgroup of persons with stroke were shown in Table 1. Stroke was detected in 206 (60.9%) of the 338 patients in the non-HHcy group and in 1312 (68.3%) of the 1920 patients in the HHcy group. In HHcy group, hypertension patients with stroke had the significantly higher FPG, SBP, DBP, and eGFR levels than those of patients without stroke, and DM was noted in 30.95% of individuals with stroke compared to 14.80% without stroke. In hypertension patients with HHcy, men were at higher risk of stroke and the age of stroke patients was statistically higher than that of patients without HHcy, as was also their exposure to environmental cigarette smoking and alcohol consumption. In HHcy group, the mean Log Hcy level in the patients with stroke was significantly higher than that in the patients without stroke.

**Table 1:** Demographic characteristics of patients with hypertension by homocysteine and stroke status. Abbreviations: HHcy, hyperhomocysteinemia; BMI, body mass index; FPG, fasting plasma glucose; SBP, systolic blood pressure; DBP, diastolic blood pressure; TC, total cholesterol; HDL-C, high-density lipoprotein cholesterol; TG, triglycerides; sCr, serum creatinine; eGFR, estimated glomerular filtration rate; Hcy, homocysteine. Continuous variables are expressed as mean ± standard deviation and categorical variables are presented as number (%). ^1^*P*-value was calculated by comparing demographic characteristics between two stroke groups and ^2^*P*-value was calculated by comparing characteristics between stroke and no stroke in patients with HHcy.

Variables	Total (n = 2258)	Non-HHcy	*P*-value^1^	HHcy		*P*-value^2^
		Stroke		Nonstroke	Stroke	
		(n = 206)		(n = 608)	(n = 1312)	
Male, no. (%)	1386 (61.38)	80 (38.83)	<0.001	376 (61.84)	873 (66.54)	0.045
Age, years	60.75 ± 8.70	58.45 ± 10.08	<0.001	61.38 ± 7.80	60.92 ± 8.95	0.256
BMI, kg/m^2^	24.69 ± 3.48	24.09 ± 3.53	0.058	25.10 ± 3.33	24.58 ± 3.50	0.082
SBP, mmHg	155.70 ± 24.56	161.72 ± 26.92	0.708	141.87 ± 16.93	162.42 ± 24.53	<0.001
DBP, mmHg	92.18 ± 14.02	95.79 ± 16.13	0.628	85.64 ± 10.09	95.26 ± 14.30	<0.001
FPG, mmol/L	6.41 ± 2.20	7.14 ± 2.99	0.008	5.93 ± 1.58	6.56 ± 2.30	<0.001
TC, mg/dL	189.12 ± 39.37	184.28 ± 38.90	0.941	198.24 ± 36.16	184.50 ± 39.73	<0.001
HDL-C, mg/dL	36.88 ± 11.71	35.67 ± 11.16	0.337	40.54 ± 11.28	34.85 ± 11.35	<0.001
Non-HDL-C, mg/dL	152.35 ± 37.65	148.61 ± 36.62	0.709	157.70 ± 34.29	149.65 ± 38.79	<0.001
TG, mg/dL	164.22 ± 86.77	163.98 ± 80.06	0.759	163.28 ± 87.55	165.99 ± 88.30	0.531
sCr, mg/dL	1.03 ± 0.20	0.96 ± 0.18	<0.001	1.07 ± 0.18	1.03 ± 0.21	<0.001
eGFR, mL/min/1.73 m^2^	72.11 ± 16.12	74.4 ± 15.99	0.448	68.45 ± 13.39	73.43 ± 17.17	<0.001
Log Hcy	1.19 ± 0.21	0.92 ± 0.07	<0.001	1.20 ± 0.16	1.25 ± 0.20	<0.001
History of, no. (%)						
Cigarette smoking	1008 (44.64)	62 (30.10)	<0.001	240 (39.47)	665 (50.69)	<0.001
Alcohol consumption	831 (36.80)	53 (25.73)	<0.001	206 (33.88)	530 (40.40)	0.006
Diabetes mellitus	596 (26.40)	78 (37.86)	0.048	90 (14.80)	406 (30.95)	<0.001
Hyperlipidemia	1101 (48.76)	96 (46.60)	0.766	315 (51.81)	626 (47.71)	0.095

### Associations between Hcy and Stroke

The associations between Hcy and risk of stroke were provided in Table 2. The univariate logistic regression analysis revealed that Hcy was associated with an increased OR of the risk of stroke in both 2258 hypertension patients (OR, 1.027; 95% CI, 1.016–1.038; P < 0.001) and 1920 H-type hypertension patients (OR, 1.026; 95% CI, 1.014–1.037; P < 0.001), after adjustment for age, sex, BMI, SBP, DBP, FPG, TC, TG, HDL-C, non-HDL-C, sCr, eGFR and history of smoking, alcohol intake, DM, and hyperlipidemia.

**Table 2:** Associations between homocysteine and risk of stroke in patients with hypertension. Abbreviations: OR, odds ratio; CI, confidence interval. Model 1: unadjusted. Model 2: adjusted for age and sex. Model 3: adjusted for age, sex, body mass index, systolic blood pressure, diastolic blood pressure, fasting plasma glucose, total cholesterol, triacylglycerols, high-density lipoprotein cholesterol, non-high-density lipoprotein cholesterol, serum creatinine, estimated glomerular filtration rate, and history of smoking, alcohol intake, diabetes mellitus, and hyperlipidemia.

	Homocysteine OR (95% CI)	*P*-value
Total	2258	
Nonstroke	740	
Stroke	1518	
Model 1	1.024 (1.015–1.034)	<0.001
Model 2	1.024 (1.014–1.033)	<0.001
Model 3	1.027 (1.016–1.038)	<0.001
H-type hypertension	1920	
Nonstroke	608	
Stroke	1312	
Model 1	1.023 (1.013–1.033)	<0.001
Model 2	1.022 (1.012–1.032)	<0.001
Model 3	1.026 (1.014–1.037)	<0.001

The stratified analyses of the associations between Hcy and stroke were presented in Table 3. In patients with hypertension, an increasing level of Hcy was associated with higher stroke risk in the following stratified factors, including sex, age (range, 60–74 yrs), BMI (range, <28 kg/m^2^), FPG ranges (<6.1 mmol/L and ≥7.0 mmol/L), SBP (range, 140–179 mmHg), DBP (range, <110 mmHg), TG (range, <150 mg/dL), and non-HDL-C (range, <190 mg/dL).

**Table 3:** Stratified analyses of the associations between Hcy and stroke in patients with hypertension. Abbreviations: Hcy, homocysteine; BMI, body mass index; FPG, fasting plasma glucose; SBP, systolic blood pressure; DBP, diastolic blood pressure; TG, triglycerides. Adjusted for age, sex, body mass index, systolic blood pressure, diastolic blood pressure, fasting plasma glucose, total cholesterol, triacylglycerols, high-density lipoprotein cholesterol, non-high-density lipoprotein cholesterol, serum creatinine, estimated glomerular filtration rate, and history of smoking, alcohol intake, diabetes mellitus, and hyperlipidemia.

	Log Hcy			P for Trend	P for Interaction
	<1.09 (n = 735)	1.09–1.23 (n = 813)	>1.23 (n = 710)		
Sex					0.001
Male, n = 1386	1	1.064 (0.751–1.506)	1.637 (1.150–2.330)	0.007	
Female, n = 872	1	1.268 (0.872–1.843)	2.389 (1.441–3.962)	0.003	
Age, years					0.537
≤ 44, n = 91	1	9.489 (1.132–79.524)	4.538 (0.740–27.848)	0.089	
45–59, n = 812	1	1.100 (0.720–1.679)	1.577 (0.970–2.564)	0.170	
60–74, n = 1275	1	1.133 (0.813–1.580)	2.021 (1.407–2.904)	<0.001	
≥75, n = 80	1	0.237 (0.020–2.864)	0.812 (0.063–10.492)	0.365	
BMI, kg/m^2^					0.613
<24, n = 967	1	1.195 (0.806–1.772)	1.873 (1.198–2.927)	0.020	
24–27.9, n = 938	1	1.217 (0.814–1.819)	2.193 (1.411–3.408)	0.001	
≥28, n = 353	1	1.048 (0.560–1.962)	1.486 (0.757–2.916)	0.447	
FPG, mmol/L					0.379
<6.1, n = 1385	1	1.318 (0.969–1.794)	2.217 (1.565–3.140)	<0.001	
6.1–6.9, n = 384	1	1.164 (0.597–2.270)	1.148 (0.587–2.242)	0.888	
≥7.0, n = 489	1	0.924 (0.495–1.724)	2.370 (1.130–4.971)	0.027	
SBP, mmHg					<0.001
<140, n = 517	1	1.182 (0.736–1.898)	1.852 (1.100–3.120)	0.056	
140–159, n = 746	1	1.153 (0.773–1.720)	1.754 (1.144–2.691)	0.027	
160–179, n = 514	1	1.866 (0.968–3.596)	2.659 (1.289–5.488)	0.026	
≥180, n = 481	1	1.276 (0.504–3.231)	6.909 (1.370–34.840)	0.063	
DBP, mmHg					<0.001
<90, n = 862	1	1.118 (0.778–1.605)	1.675 (1.125–2.495)	0.028	
90–99, n = 644	1	1.339 (0.837–2.144)	2.174 (1.286–3.675)	0.014	
100–109, n = 450	1	1.548 (0.797–3.007)	3.970 (1.697–9.288)	0.006	
≥110, n = 302	1	1.595 (0.399–6.388)	4.256 (0.693–26.137)	0.292	
TG, mg/dL					0.671
<150, n = 1208	1	1.340 (0.939–1.912)	2.346 (1.584–3.475)	<0.001	
150–199, n = 455	1	0.778 (0.436–1.388)	1.301 (0.675–2.510)	0.219	
≥200, n = 595	1	1.121 (0.681–1.844)	1.637 (0.951–2.817)	0.165	
Non-HDL-C, mg/dL					0.807
<160, n = 1346	1	1.244 (0.891–1.737)	1.947 (1.348–2.812)	0.001	
160–189, n = 584	1	1.120 (0.672–1.868)	1.973 (1.132–3.439)	0.039	
≥190, n = 328	1	1.291 (0.660–2.526)	2.056 (0.935–4.521)	0.195	

**Figure 1:**
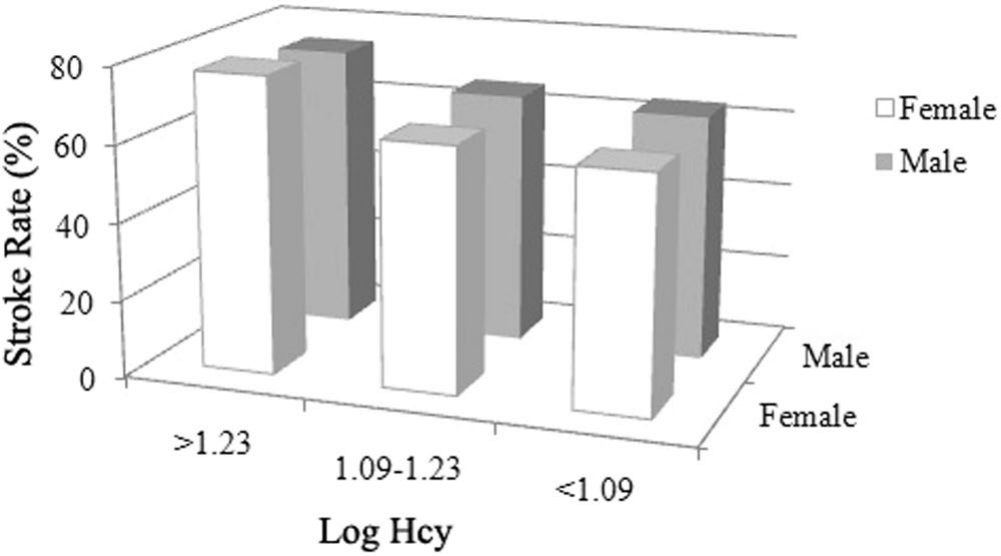
Interaction of homocysteine (Hcy) and sex on stroke risk. Among three different levels of Log Hcy in 2258 patients with hypertension, the prevalence of stroke in female (77.16%) was highest in those with Log Hcy level >1.23.

**Figure 2:**
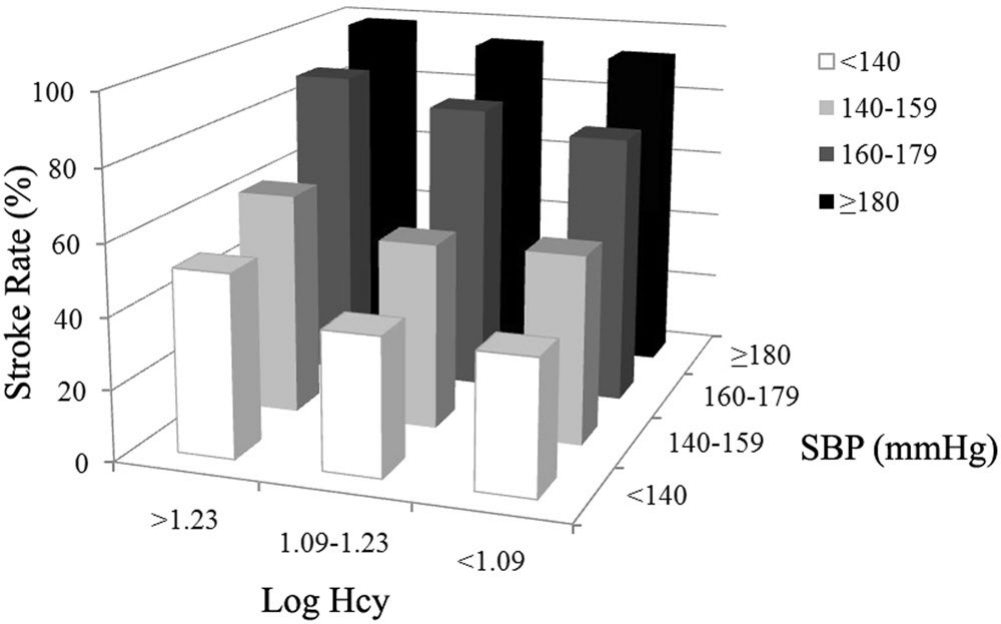
Interaction of homocysteine (Hcy) and systolic blood pressure (SBP) on stroke risk. Among three different levels of Log Hcy in 2258 patients with hypertension, those with both Log Hcy level >1.23 and SBP ≥180 mmHg were found to have the highest stroke morbidity (98.74%).

**Figure 3:**
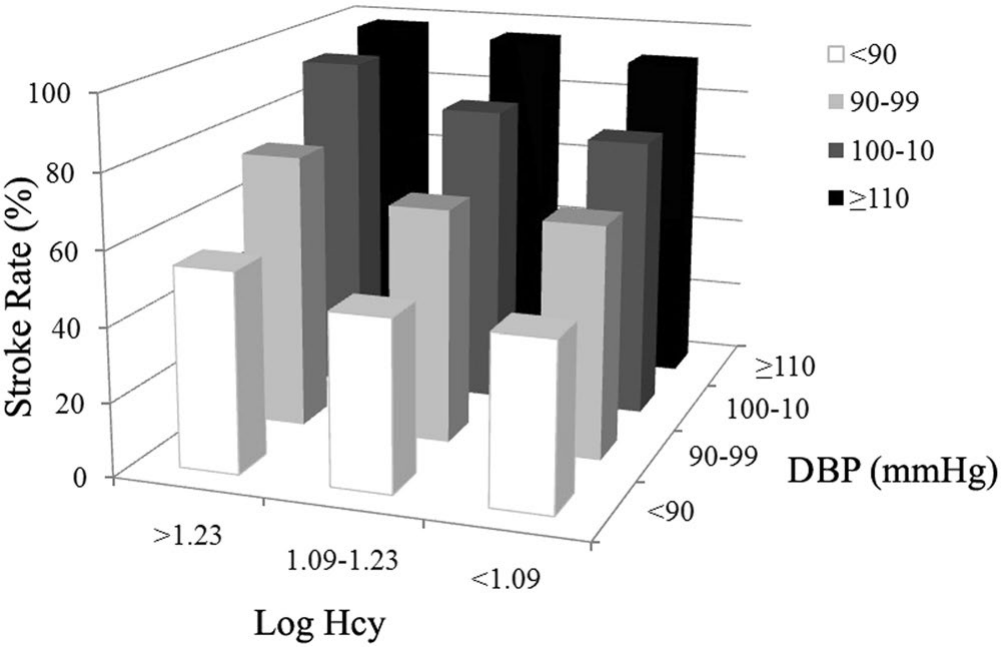
Interaction of homocysteine (Hcy) and diastolic blood pressure (DBP) on stroke risk. Among three different levels of Log Hcy in 2258 patients with hypertension, those with both Log Hcy level >1.23 and DBP ≥110 mmHg were found to have the highest stroke morbidity (98.06%).

Among the 2258 hypertension participants, three tests of interactions between Hcy and baseline characteristics of sex, age, BMI, FPG, SBP, DBP, TG, and non-HDL-C on stroke risk were statistically significant: sex (OR, 1.186; 95% CI, 1.075–1.308; P for interaction = 0.001, Fig. 1), SBP (OR, 1.183; 95% CI, 1.107–1.264; P for interaction <0.001, Fig. 2) and DBP (OR, 1.220; 95% CI, 1.149–1.295; P for interaction <0.001, Fig. 3), after adjustment for age, sex, BMI, SBP, DBP, FPG, TC, TG, HDL-C, non-HDL-C, sCr, eGFR, and history of smoking, alcohol intake, DM, and hyperlipidemia. However, other baseline characteristics of age, BMI, FPG, TG, and non-HDL-C showed no significant interactions with Hcy on stroke risk in this population.

## Discussion

In this study, we found that Hcy was an independene risk factor for stroke in both 2258 patients with hypertension and 1920 patients with H-type hypertension, after adjustment for age, sex, BMI, SBP, DBP, FPG, TC, TG, HDL-C, non-HDL-C, sCr, eGFR, and history of smoking, alcohol intake, DM, and hyperlipidemia. On the other hand, in models adjusted for the above potential confounding factors, female and elevated blood pressure significantly modified the effects of Hcy on changes in stroke risk. Risk of stroke was more strongly associated with increased SBP and DBP in patients higher than 1.23 of Log Hcy than in those 1.09 to 1.23 of Log Hcy or those lower than 1.09 of Log Hcy. There was also a significant trend according to sex, with a higher risk of stroke in female patients with Log Hcy >1.23 than in those with Log Hcy ≤ 1.23.

Effective prevention included for the control of risk factors remains the best approach for reducing the burden of stroke. Fortunately, there are enormous opportunities for preventing stroke. An analysis of data from 22 countries found that 10 potentially modifiable risk factors explained 90% of the risk of ischaemic and intracerebral haemorrhagic stroke^17^. Most studies have found hypertension and HHcy to be independent stroke predictors. In a retrospective analysis of 20 702 hypertensive patients from the China Stroke Primary Prevention Trial, visit-to-visit SBP variability was an independent predictor of primary stroke, independent of mean SBP over the follow-up period^18^. A prospective cohort study identified HHcy associated with ischemic stroke (hazard ratio, 2.18; 95% CI, 1.65–2.89). Compared with normal level of Hcy (<15 μmol/L), the hazard ratio for ischemic stroke in the highest Hcy category (≥30 μmol/L) were 4.96 (95% CI, 3.03–8.12)^5^. Our data are in line with previous studies showing a relation between Hcy and the risk of stroke. The observed OR values are comparatively modest. Because a greater prevalence of other important risk factors among hospital registers may have contributed to the generally lower OR values for the risk of stroke. The use of hospital registers is likely to have lower degree of underreporting and misclassification of potential stroke risk factors than community-based population. The validity of the database should be very high. In addition, our observation of associations in both crude and comorbidity-adjusted analyses suggests that Hcy act independently of other risk factors for stroke risk. Therefore, we strongly believe that the data are sufficiently accurate for this study purpose.

HHcy is not only an independent risk factor for stroke but also a marker of poor prognosis in stroke patients. In patients with acute ischemic stroke, white matter hyperintensity (WMH) in the periventricular and frontal areas were found to be independently associated with Hcy level^19^. Moreover, minor ischemic stroke with WMH increase experienced more recurrent cerebrovascular events. A prospective cohort study reported that the National Institute of Health Stroke Scale scores of high Hcy level group were significantly higher than those of low level group in 194 acute ischemic stroke patients after 1-week tissue plasminogen activator treatment. The high Hcy group showed obvious symptomatic intracerebral hemorrhage risk after 24 hours. In addition, the modified Rankin Scale scores of high Hcy level group after 3 months, which compared with low Hcy level group^20^. Similar findings were reported that Hcy ≥30 μmol/L increased the risk of being discharged with poor functional status in elderly patients with acute ischemic stroke, compared to Hcy < 16 μmol/L^21^. Furthermore, another prospective study that included 3799 patients with acute ischemic stroke showed that HHcy was associated with the risk of stroke-related mortality in the large-vessel atherosclerosis subtype (adjusted HR, 1.80; 95% CI, 1.05–3.07) but not in the small-vessel occlusion subtype (adjusted HR, 0.80; 95% CI, 0.30–2.12)^22^. However, results concerning the relationship between Hcy and stroke outcomes are conflicting. This study recruited 594 elderly patients with first-onset acute cerebral infarction and found that elevated Hcy was not associated with functional outcome at 3 months and 1 year after stroke^8^. Management of hypertension patients with HHcy, therefore, based on lowering blood pressure and Hcy as means to prevent stroke and improve prognosis after stroke is undisputed.

Besides an independent effect, a multiplicative effect of hypertension and HHcy substantially increases the risk of ischemic stroke^23^ and intracerebral hemorrhage^24^. To determine whether the association between hypertension and stroke might be explained through an effect on plasma Hcy, we observed a significant interaction between plasma Hcy and blood pressure on stroke risk. In the present study, we also proved the interaction between Hcy and sex on risk of stroke. In a Chinese nested case-control study with 39,165 participants who had no history of stroke, people with Hcy ≥20 μmol/L (OR, 5.1; 95% CI, 1.6–16.4) had higher risk of stroke death relative to people with Hcy <10 μmol/L. The risk of incident stroke (OR, 3.8; 95% CI, 2.3–6.4) and stroke death (OR, 3.2; 95% CI, 1.8–6.0) in people with hypertension was higher than that in people without hypertension. People with H-type hypertension had an OR for incident stroke of 12.7 (95% CI, 2.8–58.0) and an OR for stroke death of 11.7 (95% CI, 2.5–54.7)^25^. In addition, H-type hypertension is also related to the development of secondary stroke (OR, 2.990; 95% CI, 1.176–7.600)^26^. A clinical trial of 203 stroke patients, 26% had HHcy at 10 months post-stroke. The factors related with HHcy included malnutrition, heavy alcohol consumption and low level of folate and vitamin B12^27^. These factors in this study may be the potential mechanisms for how H-type hypertension might lead to recurrent stroke.

### Study limitations

There are several limitations of the present investigation. First, given that this is a retrospective, single-center, observational study, a causal relationship between elevated Hcy concentrations and stroke risk cannot be established. Second, the specific cohort (hospital based, only patients from the Department of Neurology and Cardiology) may constitute a selection bias, which may not be representative for general population suffering from stroke. Third, detailed data of when antihypertensive agents were given and for how long these remained in each patient were not included in the analysis. Thus, we were not able to analyze the effect of different antihypertensive drugs on associations between Hcy and stroke.

## Conclusions

Although Hcy appears to be associated with stroke very weakly in patients with hypertension, the effects of Hcy on stroke risk improve strong likely mediated through interactions with sex and blood pressure, and effects independent of sex and blood pressure.
